# Supplementary material for: Hypersensitivity to Distractors in Fragile X Syndrome from Loss of Modulation of Cortical VIP Interneurons
Source: J Neurosci. 2023 Nov 29;43(48):8172–88. doi: 10.1523/JNEUROSCI.0571-23.2023 (PMC10697397; doi:10.1523/JNEUROSCI.0571-23.2023)
Supplement: Table 2-1 — Table showing the demographic information for the FXS and TDC humans. Notice that individuals from both groups were matched for age. Download Table 2-1, DOCX file. [file ns-JN-RM-0571-23-s01.docx]

**Extended Data for Figure 2**. Demographic Information for FXS and TDC Groups

|  | **FXS (n=23)** | **TDC (n=22)** |
| --- | --- | --- |
| Age | 28.7 (10.0)  *11 - 45* | 30.5 (9.2) 16 – 46 |
| Deviation IQ | 44.4 (20.6)***  *18 – 91* | 109.6 (11.1) *91 – 128* |
| Vineland ABC | 48.6 (22.7) *20 – 88* | - |
| FMRP | 1.8 (2.4)***  *0 – 25* | 24.6 (4.8)  *17 – 35* |
| Sex (n, %) | 4, 84 |  |
| Mosaic (n, %) | 7, 37 |  |

Mean (Standard Deviation), *Range* given unless otherwise specified
IQ – Intelligence Quotient; ABC – Adaptive Behavior Composite; FMRP – Fragile X Messenger Ribonucleoprotein; Mosaic – Size mosaicism and/or methylation mosaicism
*** p < .001
